# For a Correct Application of the CD Exciton Chirality Method: The Case of Laucysteinamide A

**DOI:** 10.3390/md16100388

**Published:** 2018-10-16

**Authors:** Gennaro Pescitelli

**Affiliations:** Department of Chemistry and Industrial Chemistry, University of Pisa, Via G. Moruzzi 13, 56124 Pisa, Italy; gennaro.pescitelli@unipi.it; Tel.: +39-050-2219-339

**Keywords:** electronic circular dichroism, absolute configuration, molecular conformation, electric transition dipole moment, exciton coupling, TDDFT calculations

## Abstract

The circular dichroism (CD) exciton chirality method (ECM) is a very popular approach for assigning the absolute configuration (AC) of natural products, thanks to its immediacy and ease of application. The sign of an exciton couplet (two electronic CD bands with opposite sign and similar intensity) can be directly correlated with the molecular stereochemistry, including the AC. However, a correct application of the ECM necessitates several prerequisites: knowledge of the molecular conformation; knowledge of transition moment direction; and preeminence of the exciton coupling mechanism with respect to other sources of CD signals. In recent years, by using quantum-chemical CD calculations, we have demonstrated that some previous applications of ECM were wrong or based on incorrect assumptions. In a recent publication of this journal (*Mar. Drugs*, **2017**, *15*(4), 121), the ECM was employed to assign the AC of a marine metabolite, laucysteinamide A. This is a further case of incorrect application of the method, where none of the aforementioned prerequisites is fully met. Using this example, we will discuss the criteria required for a correct application of the ECM.

## 1. Introduction

The very large majority of known natural products is chiral and contains multiple centers of chirality [1]. The two enantiomers of a chiral substance most often display different biological and pharmaceutical properties [2]. As a consequence, a complete stereochemical elucidation is a common requirement in natural products discovery. A novel chiral natural compound cannot be said to be fully characterized until its absolute configuration (AC) is determined. Marine natural products are, of course, not an exception [3]. The most important method for assigning ACs is X-ray structure determination [4]. Unfortunately, it is restricted to crystalline materials of compounds exhibiting enough strong anomalous dispersion. Alternatively, a wide family of chiroptical techniques exists specifically designed to analyze chiral nonracemic substances [5,6]. Of these, electronic circular dichroism (ECD, or simply CD) remains the most popular one in the field of natural products discovery [7], although vibrational CD (VCD) and Raman optical activity are gaining progressive interest [8,9]. Currently, the most common approach to assign AC by ECD or VCD analysis consists in the comparison between experimental and quantum-chemical calculated spectra [10]. However, there are several other means for interpreting ECD spectra to be used for AC determination [7,11].

The exciton chirality method (ECM) has been for a long time the most employed approach for assigning the AC of natural products by ECD spectra [12]. First developed for compounds containing a diol moiety convertible into a bis(benzoate) [13], it has then been applied to hundreds of natural compounds with very diverse skeletons and functionalities [14,15]. In a nutshell, the essence of the method is the following: a chiral molecule containing two separate chromophores with electric-dipole-allowed electronic transitions—if properly arranged with respect to each other—displays a CD spectrum containing a so-called exciton couplet, that is, two bands of opposite sign and similar intensity, centered around the chromophores UV maxima. The couplet sign (defined as that of the long-wavelength component) correlates with the absolute angle of twist defined by the two transition dipoles: a positive couplet indicates that the two transition dipoles define a positive chirality, that is, when viewed along the line connecting the dipoles, one would need a clockwise rotation to move from the dipole in the front onto that in the back. Obviously, the dipole chirality is dictated by the AC, but it also depends on the molecular conformation. In many favorable cases, just looking at the CD spectrum will yield the AC assignment, provided that a good molecular model is utilized. The most important quality of the ECM is its ease of application; it is also very robust, because it is based on a well-established theoretical basis [6,12]. These two facts render the ECM still popular, in the present computational era, to assign the AC of natural products including marine-derived ones, as demonstrated by a few recent references selected from this journal [16,17,18,19,20,21]. The method is so useful that when a compound lacks the necessary chromophores, it may be convenient to introduce them covalently or noncovalently to apply the ECM [15,22], rather than resorting to a different approach.

Any method for structural determination, however robust and efficient it may be, relies upon a correct application which meets all necessary prerequisites and hypotheses. For the ECM, the latter includes the knowledge of the molecular conformation and of transition moment directions, and confidence that the ECM dominates the CD spectrum. Strictly speaking, the ECM should not be applied unless all these pieces of information are safely known; otherwise, its application is not justified and may lead to incorrect AC assignments. We have demonstrated that incautious applications of the ECM still happen to appear in recent literature reports [23,24,25]. In this contribution, we will examine a recent example of AC assignment of a marine product published in this journal, laucysteinamide A (**1**) [17]. We will first present an independent interpretation of the CD spectrum of **1** by means of density functional theory (DFT) calculations. Based on our analysis, we will then criticize the previous assignment to emphasize which are, and how to meet, the necessary criteria for a safe and accurate application of the ECM.

## 2. Results

**Scheme 1:**
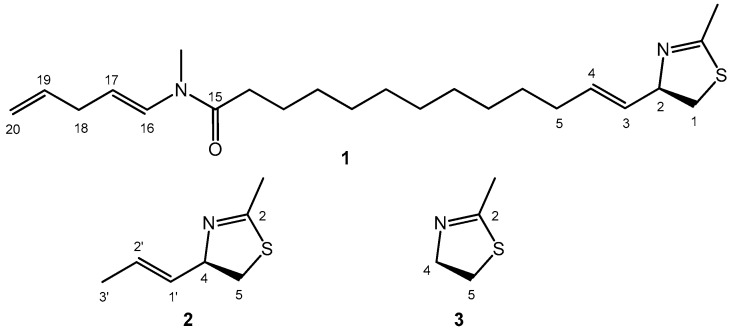
Molecular diagram of (2*R*)-laucysteinamide A (**1**), of its truncated analog (2*R*)-2-methyl-4-(prop-1-en-1-yl)-4,5-dihydrothiazole (**2**), and the model chromophore 2-methyl-4,5-dihydrothiazole (**3**).

**Figure 1:**
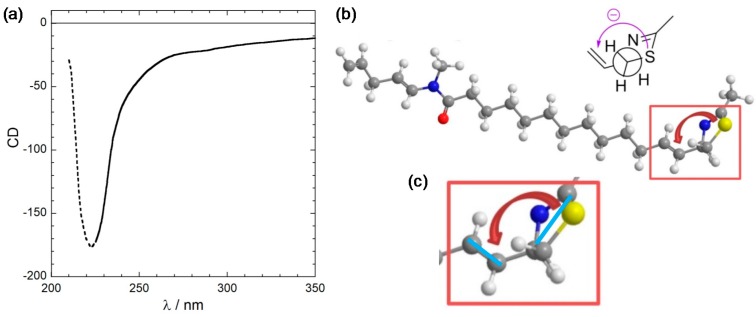
(**a**) CD spectrum of laucysteinamide A (**1**) recorded in CH_2_Cl_2_. Measurement conditions (concentration and path length) were not given, thus the *y*-axis units are undetermined. The trend below 225 nm (dotted part) is probably an artifact because of the solvent cutoff. (**b**) Exciton chirality reported in the original publication. Notice the absence of transition dipole moment directions as well as the unclear perspective. (**c**) Zoom of the relevant molecular portion where we added the most likely transition dipole moment directions (light blue bars) as suggested by the curved arrows. Panel (**a**) is adapted and (**b**,**c**) are reproduced from ref. [17].

Laucysteinamide A (**1**, Scheme 1) is a cytotoxic compound isolated from *Caldora penicillate* [17]. During its structural elucidation, the AC at the C-2 chirality center was established by recording the CD spectrum and applying the ECM. The CD spectrum of **1** contains a single negative band above 200 nm (Figure 1). Although the quality of the experimental spectrum is poor (see legend of Figure 1), we can confidently assume the presence of a negative CD band above 210 nm or at least a tail due to the same band above 220 nm. This band was assigned by the authors to the long-wavelength component of a “couplet” emerging from the nondegenerate exciton coupling between the C-3/C-4 alkene (absorbing below 200 nm) and the thiazoline chromophore (absorbing above 200 nm).

Exciton coupling is said to be degenerate (DEC) when it occurs between equivalent transitions of two identical chromophores. Conversely, nondegenerate exciton coupling (NDEC) arises between different chromophores [6,12,26]. While DEC generates a couplet centered around the single chromophore transition, NDEC generates two distant CD bands, still with opposite signs and similar intensity, each centered in correspondence with one chromophore transition. In the extreme situation, one of the two transitions involved in NDEC may occur at too high energy (or short wavelengths, <200 nm) to be observed. This is the case of the alkene π–π* transition, which is polarized along the double-bond direction and normally occurs at 195 nm. For instance, in the so-called allylic benzoate method [12,27], the alkene π–π* transition is excitonically coupled to the benzoate π–π* transition occurring around 230 nm. The long-wavelength component of the “couplet” will then appear at 230 nm, while the short-wavelength component will be often masked by the solvent cutoff.

Although not openly stated by the authors [17], the ECM analysis of laucysteinamide A (**1**) was possibly inspired by the similarity with allylic benzoates, with the thiazoline ring replacing the benzoate. As a matter of fact, the CD section in the original paper is very concise and many details were uncovered. For example, the authors apparently neglected the presence of the other two chromophoric moieties, the enamide from C-15 to C-17, and the further alkene at C-19/C-20 (Scheme 1). Though not explicitly justified, this is in fact a correct procedure because these chromophores are separated from the C-2 chirality center by a long flexible chain, therefore their contribution to the CD spectrum is negligible. In other words, one can safely consider, instead of the whole molecule of **1**, its truncated analog **2** (Scheme 1). This truncation approach has been successfully employed in CD analyses [28], and we will use it again here.

Apart from the missing details and poor experimental spectrum, the reported application of the ECM to laucysteinamide A (**1**) is flawed by many mistakes and oversights, and must be considered incorrect. As we shall see below, the assigned absolute configuration (2*R*)-**1** is the right one, although its correct assignment [17] must be consider fortuitous. A list of the most important issues, which will be analyzed more in detail in the Discussion section, is the following:

1. The nature and polarization of the transition of the thiazoline chromophore responsible for the CD band above 220 nm were not discussed; it was not clear whether this is a π–π* transition, namely, the kind of transition which is expected to be involved in exciton coupling;
2. In the drawing of **1** used to establish the chirality associated with the NDEC, the direction of the transition moment allied with the thiazoline chromophore was not shown, and an ambiguous viewpoint was assumed;
3. No conformational analysis of **1** was run, or at least it is not reported; ECM was applied to a single conformer whose relative population is unclear.

These inconsistencies prompted us to run an independent analysis of the CD spectrum of **1**, by means of a well-established computational procedure based on DFT and time-dependent DFT (TDDFT) calculations [7,29]. The procedure was run on the truncated analog (*R*)-**2** and the model for the thiazoline chromophore **3** (Scheme 1), and the results of the various steps are reported in the following sections.

### 2.1. Electronic Transitions of the Model Thiazoline Chromophore ***3***

The ECM applied to laucysteinamide A (**1**) invoked the exciton coupling between an alkene and a thiazoline chromophore [17]. While the former is well known and its participation to exciton coupling mechanism well documented, like in the allylic benzoate method mentioned above [12,27], no previous application of ECM to the thiazoline chromophore is described in the literature, to the best of our knowledge. CD analyses of chiral thiazoline-2-thiones have been reported in several instances [30,31], but this is indeed a totally different chromophore. Also different are chromophores like luciferins, subject to much attention [32], where the thiazoline ring is part of an extended chromophoric system. On the contrary, little is known on the simpler thiazoline chromophore which is of interest for the current ECM application.

To fill the gap, we first investigated compound **3** as model chromophore of the thiazoline ring in **1**. Its geometry was optimized at ωB97X-D/6-311+G(d,p) level and TDDFT calculations were run at CAM-B3LYP/ def2-TZVP level, yielding the data shown in Table 1 and summarized below:

- the first calculated transition is a magnetic-dipole-allowed sulfur-centered n–σ*;
- the second calculated transition is a magnetic-dipole-allowed nitrogen-centered n–π*;
- the third calculated transition is a π–π* transition; this is an electric-dipole-allowed transition associated with a rather weak electric transition dipole (oscillator strength *f* ~ 0.05).

**Figure 2:**
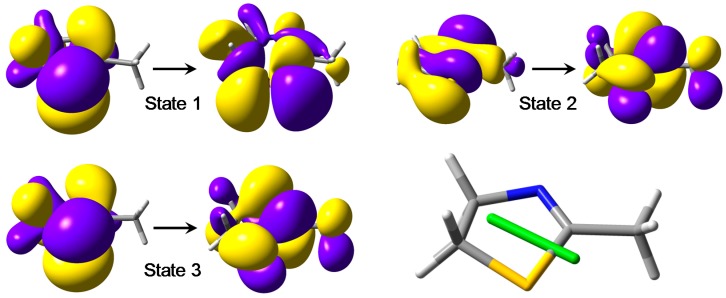
NTO plots of the first three transitions of **3** calculated at CAM-B3LYP/def2-TZVP level. The molecular orientation is shown in the lower right panel, also displaying the electric transition dipole for the 3rd transition (π–π*).

In Figure 2, the natural transition orbitals (NTO) [33] for the three transitions are depicted. This approach offers a compact representation of the electronic transition densities associated with the various transitions. The main character of each transition is described as a single excitation from an occupied to a virtual NTO. Notice how, for the first two transitions, the occupied → virtual excitation describes a rotation of the transition density, which is typical of magnetic-dipole-allowed transitions. The third excitation describes instead a translation of the transition density, which is typical of electric-dipole-allowed transitions like π–π* ones. The direction of the transition dipole for the π–π* transition is drawn in Figure 2; it is oriented approximately as the C-5/S bond of **3**.

### 2.2. Conformational Analysis and Geometry Optimizations of Truncated Model ***2***

**Figure 3:**
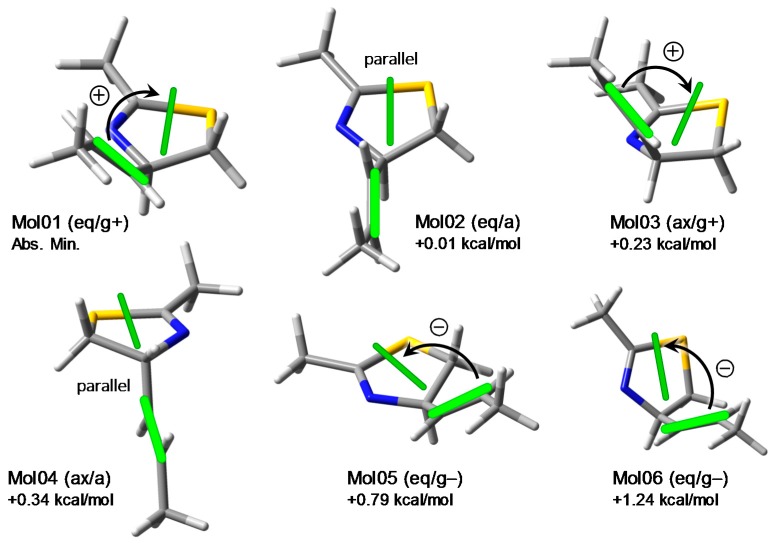
Conformers and relative energies of model compound **2** obtained after ωB97X-D/6-311+G(d,p) geometry optimization, and exciton chirality defined by the two transition dipole moments (green bars, for alkene and thiazoline π–π* transition, see text). Legend for the notation: ax/eq, pseudo-axial/equatorial position of the propenyl group; a/g+/g−, anti/gauche+/gauche− relation between H-4 and H-1′.

The conformation of the model compound **2** was investigated by molecular mechanics conformational analysis and DFT geometry optimizations. All possible conformers of (*R*)-**2** were generated by a systematic conformational search using the Merck molecular force field (MMFF). The structures were then optimized with DFT at ωB97X-D/6-311+G(d,p) level. Six low-energy conformers were obtained due to the rotamerism around the C-4/C-1′ bond and the five-membered ring puckering. They can be described using the anti/gauche notation for the reciprocal orientation of H-4 and H-1′, and the pseudo-equatorial/axial position of the propenyl group attached at C-4. The structures and relative energies are displayed in Figure 3; notice the small energy differences between the various conformers.

These results suggest that laucysteinamide A (**1**) may also exhibit a pronounced flexibility around the C-2/C-3 bond and in the thiazoline ring, although the relative populations between the various conformers may differ from those of its truncated model **2**. Such a flexibility is crucial for the exciton coupling mechanism because the noticed motions affect directly the relative orientation of the two chromophores supposed to be involved in the ECM. This fact can be appreciated by looking at the green bars shown in Figure 3 representing the transition dipole directions for the alkene π–π* (polarized along the C=C bond) and the thiazoline π–π* transition. This latter corresponds to the third calculated excited state for model chromophore **3** discussed above. It can be seen that, for a given axial/equatorial conformer of **2**, the three rotamers around the C-4/C-1′ bond exhibit variable chirality defined by the transition dipoles, either positive, negative, or negligible (when the two dipoles are approximately parallel). It is impossible to establish with certainty the resulting sign of the exciton chirality on a qualitative ground (i.e., without quantitative exciton coupling calculations) [34], though one might expect a dominant positive chirality. Regrettably, in the original publication, a negative chirality was inferred (see Figure 1 and Section 3.3 below).

### 2.3. CD Calculations on Truncated Model ***2*** and Transition Analysis

**Figure 4:**
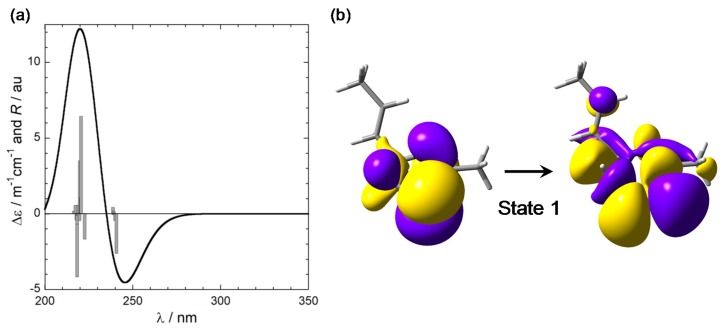
(**a**) Calculated CD spectrum of truncated model **2** at CAM-B3LYP/def2-TZVP level. The vertical bars represent the rotational strengths *R* calculated for the various conformers. (**b**) NTO plot of the first transition of **2** for the lowest-energy conformer (Mol01 in Figure 3).

To simulate the CD spectrum of model compound (*R*)-**2**, which retains the same chromophoric portion as (*R*)-**1**, TDDFT calculations were run on the six conformers described above using B3LYP and CAM-B3LYP functionals and def2-TZVP basis sets, including the IEF-PCM solvent model for dichloromethane. CAM-B3LYP/def2-TZVP results are described below; B3LYP functional led to consistent results. The six calculated spectra were averaged using internal energies estimated at ωB97X-D/6-311+G(d,p) level. The final weighted-average-calculated CD spectrum for (*R*)-**2** is shown in Figure 4. In the wavelength region >230 nm, it displays a negative CD band which corresponds to the experimental band >220 nm. A second positive band appears between 200 and 230 nm, which is impossible to compare with the experimental spectrum because of the solvent cutoff. Therefore, we must limit our discussion to the first band. We can confirm, at least on the basis of this limited spectral comparison, that the absolute configuration of laucysteinamide A (**1**) is (2*R*). It must, however, be stressed that AC assignments based only on a single band are discouraged [11], and the present case is especially critical because the experimental band is poorly defined (Section 2.1).

In all relevant conformers of **2**, the first calculated transition around 240 nm, responsible for the “diagnostic” CD band, is essentially a magnetic-dipole-allowed sulfur-centered n–σ* transition, like in the model chromophore **3**; in addition, some character of charge transfer from the thiazoline to the alkene can be recognized. The NTOs calculated for the lowest-energy conformer of **2** (Mol01 in Figure 3) are depicted in Figure 4. In conclusion, TDDFT calculations on model compounds **2** and **3** demonstrate that the negative band (or tail) appearing in the CD spectrum of laucysteinamide A (**1**) is not a component of a “couplet” arising from nondegenerate exciton coupling. Though centered on the thiazoline ring, it is not an electric-dipole-allowed π–π* transition but a magnetic-dipole-allowed sulfur-centered n–σ* transition.

## 3. Discussion

Having analyzed the conformation, CD spectrum, and electronic properties of the truncated model **2** in detail, we shall now examine the major pitfalls and inconsistencies found in the reported application of the ECM to laucysteinamide A (**1**). This exercise will help in recapitulating the criteria for a safe and accurate application of the ECM.

### 3.1. Missing Conformational Analysis

The published report on the AC assignment of laucysteinamide A (**1**) contains no detail on its conformation [17]. Apparently, no conformational analysis of **1** was run. The procedure used to obtain the molecular model used to establish the transition moment chirality is briefly described: «Computational molecular models […] were subjected to energy minimization with MOPAC software»; then, a single structure was considered for the ECM application (Figure 1). One must conclude that only a single conformer was taken into account, with unspecified relative population. The validity of this approximation is contradicted by experimental and computational data. Among the few available NMR data of **1**, we noticed the following ^3^*J*_HH_ coupling constants relative to H-1, H-2, and H-3: ^3^*J*_H1a,H2_ = ^3^*J*_H1a,H2_ = 8.4 Hz; ^3^*J*_H2,H3_ = 6.6 Hz. The single conformer used for the ECM (Figure 1) has a pseudo-axial H-2, which would lead to substantially different couplings with the pseudo-axial/equatorial H-1a and H-1b. Moreover, the expected ^3^*J*_H2,H3_ vinylic coupling constant would be 11–12 Hz for anti-oriented and 3–4 Hz for gauche-oriented H-2 and H-3, respectively [35]. Thus, the experimental values of the diagnostic ^3^*J*_HH_’s indicate an average situation allied with a large conformational freedom in the ring conformation and around the C-2/C-3 bond. The results of our conformational study on truncated model **2** (Section 2.2) also confirm the existence of multiple conformers. More importantly, we have demonstrated that the exciton coupling may vary sizably in intensity and even in sign from conformer to conformer. Any exciton-coupled CD, if observed at all, would be the weighted average of all possible situations, yielding an overall couplet difficult to predict.

The impact of conformation on exciton-coupled CD spectra has been demonstrated in several instances [36,37,38], and it is just a specific case of the well-known sensitivity and dependence of CD spectra on molecular conformation [39]. The first criterion for a correct ECM application is then: *run a thorough conformational analysis and estimate the exciton coupling for the various conformers*. The *ECM can be safely applied only if a dominant sign of the exciton chirality is apparent* among the most populated conformers. As a corollary, we recommend that the *conformational analysis is run by a combination of experimental and computational methods* [39,40].

### 3.2. Nature of the Transition Involved

In the published report on the AC assignment of laucysteinamide A (**1**), the NDEC between the alkene and thiazoline chromophores was invoked [17]. The authors noticed that the «CD spectrum of compound **1** showed a negative local maximum at 223 nm, corresponding to the thiazoline chromophore», while «the maximum expected from the C-3/C-4 alkene would be around 190 nm, but was not observed in the spectrum due to solvent absorptions». This is analogous to the allylic benzoate method recalled above (Section 2.1). As a matter of fact, the nature of the specific transition of the thiazoline chromophore involved in the NDEC was not discussed [17]. Since π–π* transitions are normally considered in ECM, because of their electric-dipole-allowed character, one might expect that the authors assumed the existence of a π–π* transition around 220 nm. In addition, the direction of the electric dipole moments should be known prior to ECM applications, because it is the reciprocal arrangement between the transition dipoles which dictates the sign of the CD spectrum. Most of the aromatic or π-conjugated chromophores usually encountered in ECM, for example, *p*-substituted benzoates [12,13], naphthalene [41,42], enones [43], etc., have a well-known electronic structure and the direction of the relevant transition dipoles is certain. In many other cases, the transition dipole polarization may be inferred from the symmetry properties of the chromophores. For example, an all-*trans* polyene chain, or a polyenone, has an effective cylindrical symmetry for which the relevant π–π* transitions are necessary polarized along the chain direction; ECM applications are therefore straightforward [16]. On the contrary, the thiazoline ring lacks any element of axial symmetry, therefore π–π* transitions can be, in principle, oriented in any direction in the plane. It appears that this chromophore is not ideal at all for ECM applications. Still, in this situation, one may resort to running excited-state calculations to study the chromophore and to assess the direction of the transition moments. Apparently, such necessity was overlooked by the authors [17].

As a result of our TDDFT calculations on **2** and **3**, we already demonstrated that the transition responsible for the CD band >220 nm is not an electric-dipole-allowed π–π* transition, which could give exciton coupling with the alkene π–π* transition. The first calculated transition is instead a magnetic-dipole-allowed sulfur-centered n–σ* transition. This means that the diagnostic band in the CD spectrum of **1** is not due to the exciton coupling mechanism but to other mechanisms capable of generating CD signals, in this case, those typically associated with sulfur n–σ* transitions [44].

In summary, the *ECM should be applied to well-known chromophores* for which *the direction of the transition dipole moment is established*. “New” chromophores require their electronic structure to be studied independently. *ECM can be invoked only for electric-dipole-allowed transitions*, in most cases, π–π* transitions of aromatic chromophores or extended π-conjugated chromophores. In all cases, moreover, one must be confident that the *ECM is truly responsible for the observed CD spectrum*, and interference from other sources of CD signals may be excluded. This is especially important for NDEC, which is intrinsically weaker than DEC and generates weak CD “couplets” easily overruled by other sources of CD signals [24]. Polavarapu provided a useful formula to estimate the strength of CD signals associated with NDEC; see p. 360 in Ref. [6].

### 3.3. Viewpoint Used to Establish the Transition Moment Chirality

**Figure 5:**
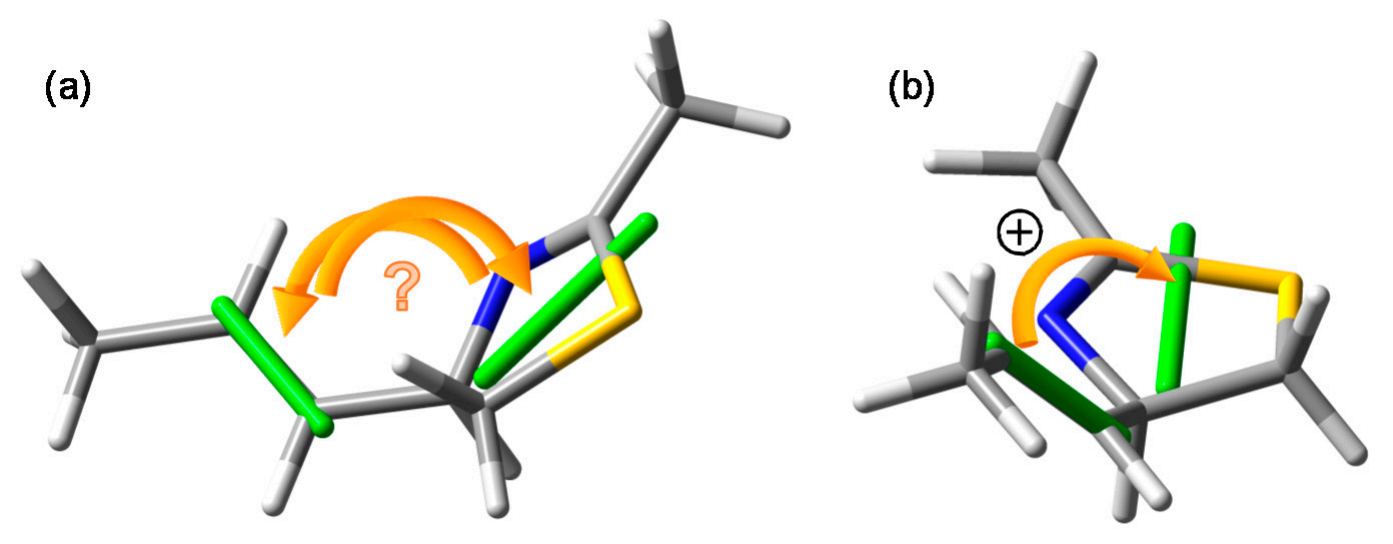
Lowest-energy conformer of model compound (*R*)-**2** seen in two different perspectives. Viewpoint (**a**) is the same assumed in the original publication (see Figure 1c), where a negative chirality was seen for (*R*)-**1** [17]. The chirality defined by the transition moments is in fact ambiguous. Viewpoint (**b**) offers a better perspective of the actual positive chirality.

When assessing the exciton chirality defined by the two transition dipole moments, one must follow a few steps: (a) generate one (or more) meaningful molecular model representative of the most relevant conformer(s), see Section 2.2; (b) depict it in an intelligible way with one chromophore clearly placed in the front and the other in the back; (c) draw the transition dipole moments with their correct direction in the middle of the respective chromophores, see Section 2.1; (d) establish the sense of rotation (clockwise or anticlockwise), which is conceptually necessary to move the dipole in the front onto that in the back. Figure 3 was conceived according to these prescriptions. This is instead not the case for the drawing used to establish the exciton chirality in laucysteinamide A (**1**) [17], reproduced in Figure 1. We have already noticed the missing transition moment directions (Section 3.2). More importantly, even admitting the most plausible directions, the viewpoint adopted by the authors remains ambiguous, as one cannot safely establish which chromophore lies in front and which in back. This problem can be appreciated using our molecular model for the lowest-energy DFT conformer of (*R*)-**2** (Mol01 in Figure 3), which corresponds to the conformation used by the authors for (*R*)-**1**. In Figure 5a, we plot this molecular model, on which the correct transition moments are projected, using the same viewpoint of the original drawing: it is impossible to establish the exciton chirality unambiguously. This is instead possible by plotting the same molecular model more meaningfully as in Figure 5b: once the correct viewpoint is assumed, the chirality defined by the transition dipole moments turns out to be positive. Regrettably enough, this is opposite to the negative chirality inferred in the original publication for (*R*)-**1** (Figure 1) [17].

One can expect that such a trivial error is unique; unfortunately, this is not unprecedented [23]. The take-home message is that *the molecular model used to establish the transition moment chirality must be drawn in an unambiguous way*, that is, with one chromophore clearly placed in the front and the other in the back. The *less ambiguous viewpoint is along the line connecting the middle of the two chromophores*, although often this does not offer a good perspective of the molecule. A more extensive treatment of this latest issue may be found elsewhere [23].

## 4. Materials and Methods

Molecular mechanics and preliminary Density Functional Theory (DFT) calculations were run with Spartan’16 (Wavenfunction, Irvine, CA, USA, 2016) with default parameters, default grids, and convergence criteria; DFT and Time-Dependent DFT (TDDFT) calculations were run with Gaussian16 (Gaussian. Inc., Wallingford, CT, USA) [45] with default grids and convergence criteria. Conformational analyses were run with the Monte Carlo procedure implemented in Spartan’16 using Merck molecular force field (MMFF). All structures obtained thereof were optimized with DFT at the ωB97X-D/6-311+G(d,p) level in vacuo. TDDFT calculations were run with B3LYP and CAM-B3LYP functionals and def2-TZVP basis set, and included the polarizable continuum model (PCM) for CH_2_Cl_2_ in its Integral Equation Formalism (IEF) formulation. Average ECD spectra were computed by weighting ECD spectra (calculated for each conformer) with Boltzmann factors at 300K estimated from DFT internal energies. CD spectra were plotted using the program SpecDis [46,47], applying the dipole-length formalism for rotational strengths; the difference with dipole-velocity values was negligible. Natural transition orbitals were plotted with GaussView6 (Semichem, Inc., Shawnee Mission, KS, USA).

## 5. Conclusions

The exciton chirality method (ECM) is a powerful, robust, and rapid method for assigning the absolute configuration (AC) of natural products, provided that they contain two or more chromophores giving rise to electric-dipole-allowed electronic transitions. The two chromophores need to be (relatively) close in space but not directly conjugated with each other or mutually involved in charge-transfer transitions. To apply the method, the CD spectrum should display a *diagnostic CD couplet*, or at least its long-wavelength component should be clearly identified. Henceforth, by looking at the *molecular structure*, one must establish the chirality defined by the two *transition dipoles*, and correlate it with the experimental CD sign.

At first sight, one may expect that the above facts can be simply assessed by looking at the molecular diagram and spectra. In fact, crucial details are hidden within the italicized words. Looking at the *molecular structure* means that this latter must be known, that is, the molecular conformation must have been safely determined and all the important conformers identified—namely, those most contributing to the experimental CD spectrum. Establishing the chirality defined by the two *transition dipoles* means that their exact direction within each chromophore must be known in advance, or must be established before applying the ECM. Finally, identifying a *diagnostic CD couplet* implies that the CD spectrum is dominated by the exciton coupling mechanism, while other sources of CD signals are absent or negligible. None of these pieces of information is trivial and their importance is easily overlooked by nonexperts. Not surprisingly, then, they are the main sources of incorrect applications of the ECM. Laucysteinamide A (**1**) seems to be a very unfortunate case where none of the above prescriptions was met. It lends itself as a negative but instructive example of the major traps which can be encountered on the way to assigning ACs by the ECM. The present critical analysis was mainly intended to stress the necessary prerequisites and criteria for a safe application of the ECM, and to guide on how to achieve them.

## Figures and Tables

**Table 1:** Main parameters for the first three electronic transitions of **3** calculated at CAM-B3LYP/def2-TZVP level.

State	Energy (eV)	Wavelength (nm)	Oscillator Strength
1	5.208	238.1	0.0018
2	5.697	217.6	0.0040
3	5.775	214.7	0.0495
